# Gender and Child Behavior Problems in Rural Nepal: Differential Expectations and Responses

**DOI:** 10.1038/s41598-019-43972-3

**Published:** 2019-05-21

**Authors:** Julia A. Langer, Julia V. Ramos, Lajina Ghimire, Sauharda Rai, Brandon A. Kohrt, Matthew D. Burkey

**Affiliations:** 1 Johns Hopkins Bloomberg School of Public Health, Baltimore, USA; 2 Johns Hopkins University School of Medicine, Baltimore, USA; 3 Transcultural Psychosocial Organization - Nepal, Kathmandu, Nepal; 4 University of Washington, Jackson School of International Studies, Seattle, USA; 5 Department of Psychiatry and Behavioral Sciences, The George Washington University, Washington, USA; 6 Department of Psychiatry, University of British Columbia, Vancouver, Canada

**Keywords:** Human behaviour, Epidemiology

## Abstract

Whereas epidemiologic studies consistently identify different rates and types of problematic behavior in boys and girls, there has been little research examining the ecocultural context in which these gender differences in child behavior problems develop, especially in non-Western settings. This qualitative study in rural Nepal explored how behavioral expectations differed based on gender role, gender discrimination, inequity, and treatment of children based on their gender identity. We conducted semi-structured interviews with a total of 14 parents, school workers, and community leaders from a village in rural Nepal. Interview transcripts were coded by two authors using predetermined and emergent codes to identify expectations, behavior problems, and responses to behavior problems, stratified by gender. Authors then arranged codes into categories based on emergent themes. Four major themes in the interviews were identified: (1) self-reported gender non-bias; (2) differentiated role expectations; (3) gender, “goodness”, and differential thresholds for problem behaviors; and (4) boys and girls require different responses for misbehavior. Results from our study in Nepal reflect nearly universal models of gender differences in behavior. Of particular importance in South Asia, patrilocal marital practices were used to frame gender differences in expectations. To protect girls’ future potential to marry, local cultural practices provide girls with lesser opportunities and less cultural space to conduct themselves in a disruptive manner than boys. Greater understanding of differential expectations and responses to disruptive behaviors by gender will be important for culturally-appropriate equitable programming in child development.

## Introduction

Epidemiologic studies consistently identify gender differences in the prevalence and patterns of behavior problems^1,2^. In epidemiologic reports and disorder definitions, these gender differences are often left unexamined or interpreted through the lens of biological determinism^1,3,4^. Closer examination of the social construction of gender may elucidate interactions between ecocultural factors and child development that lead to differences in perceptions of similar behaviors in boys versus girls. Further study is important to better understand gender discrepancies in psychosocial outcomes later in development and to tailor interventions to child behavior issues using gender and therefore the ecocultural context of child development as a guide. This qualitative case study examines how behavioral expectations among Nepali caregivers differed based on gender role, gender discrimination, inequity, and treatment of children based on their gender identity.

Girls and boys are expected to participate in society differently. Girls are often expected to be more feminine and to participate in different social spheres than their male counterparts. In Nepal, like many patriarchal cultures, it is recognized that girls are marginalized, educated at lower rates, and many are forced to marry early or are trafficked for commercial sex work^5–7^. Girls tend to communicate their femininity through expressions of fearfulness or the avoidance of feared objects or situations, and remaining quiet. Girls also occupy the domestic sphere more than boys and are often required to complete substantially more housework than boys^8,9^. Conversely, boys are expected to be masculine by confronting fearful situations and limiting their expressions of emotions. Boys and men have a higher status in patriarchal societies and are more likely to be perceived as good leaders, while the inverse is true for girls and women^10,11^.

These gender roles and expectations placed on boys and girls have tangible impacts^12^. To elucidate the impact, we will focus on masculinity and violence. The masculinity encouraged in boys serves as an unchecked expression of power, which increases their propensity towards delinquency, retaliation towards other boys and violence towards girls^13–16^. Unsurprisingly, the propensity towards expressing violence is lower in girls and women. Girls have been reported to exhibit more relational aggression (i.e. attempting to harm one’s relationship with others), while boys are more likely to exhibit physical aggression^17^. For example, a study conducted in rural Nepal found that women are more likely to use alcohol to cope when they experience stress or psychological distress and less likely to use violence, compared to men^18^. Because violence is linked to masculinity and therefore a form of power, some of the most vulnerable Nepali girls would become child soldiers as a form of empowerment when access to traditional vehicles of empowerment like education were nearly nonexistent^19^. In 2016, the Demographic and Health Survey found 17.7% of Nepali girls age 14–19 reported being survivors of physical or psychological violence and 4.0% reported experiencing sexual violence, usually by a male partner or spouse^20^. The risk of girls and women experiencing any form of violence decreases with increasing levels of education, where roughly 34% of women with no education experience physical violence compared to 8% of women who graduated from secondary school^20^. Until recently, educational opportunities were primarily reserved for males, although the right to education for girls is increasingly being recognized in Nepal, and the number of girls going to primary schools has increased by 3.5% per year^21^. Despite increasing school enrollment, the domestic workload of girls often prevents regular school attendance and continuing education, thus perpetuating the high rates of violence and disempowerment of girls^17^.

Globally, boys are found to have higher rates of behavior problems than girls in nationally representative surveys and cohort studies. These studies often lack discussion regarding masculinity and its tangible impacts on girls and boys such as the example of violence above. Despite high rates of delinquency in boys, boys later as men are often overrepresented in Nepali leadership while girls are excluded later as women on family, community, and institutional levels^1,2,7^. Objectivist interpretations of these differences, however, are problematic for several reasons—they reaffirm gender stereotypes and consider gender to be a primary cause of behavior without attending to its symbolic meaning or social context. Along these lines, Gaines^3^ has argued that psychiatric diagnostic categories found in texts such as the *Diagnostic and Statistical Manual* (DSM) express an “underlying cultural psychology,” in which gendered behavioral problems are constructed as psychiatric disorders and thus assumed to be biologically associated with gender. Instead, a constructivist evaluation of the interface between culture, gender, and behavior problems is needed in order to place these epidemiologic findings in context^5^.

Understanding and effectively addressing behavior problems in ways that are culturally relevant and avoid perpetuating gender-based inequity requires human development theories that are not only informed by human biology, but also by anthropology^22–24^. Definitions of child behavior that do not account for gender differences in expectations lack validity and reify gender constructs. For example, in DSM-5, definitions of disruptive behavior disorders note that gender may influence the threshold for diagnosis, depending on normative levels of behavior problems; however, the definition does not discuss how to treat situations in which the same behavior may be acceptable in boys but viewed as problematic in girls^4^. When pathologized behavior is not associated with distress or impairment, or recognized in the local context as problematic, there is a risk of what Kleinman refers to as a “category fallacy”^25^. Gender inequity is a deeply rooted in cultural systems, which, in turn, are perpetuated across generations beginning in the context of the household during childhood^26,27^. Qualitative analyses of the contextual factors such as gender expectations made by family and community standards help demonstrate the social construction of “problematic” child behavior, and thus would highlight its effect on child development and child and adult mental health^23,28–31^.

## Theoretical Framework

In this study, we draw upon a constructivist epistemology in order to understand how knowledge about child behavior problems is created in particular social contexts^32^. A constructivist epistemology rejects objectivists’ claims to reality and assumes that meaning or knowledge are not discovered but instead are constructed or created. In this study, we utilize symbolic interactionism as our primary theoretical perspective to understand how social interactions create meaningful interpretations of gender and child behavior^33^. Symbolic interactionism is a theory that grounds our assumptions about how meaning is made and how behavior is shaped, and guided our selection of the research methodology and analytic approach used in this study^32^.

While previous studies have evaluated the impact of specific parental investments in health, education, and nutrition on children’s behavior and development, this study considers parental ethnotheories as a major factor influencing parents’ interpretations of (i.e. as acceptable or problematic) and responses to child behavior. We draw upon Super and Harkness’ definition of parental ethnotheories as “beliefs concerning the nature and needs of children, parental and community goals for rearing, and caretaker beliefs about effective rearing techniques (pg. 556)”. As such, we envision cultural differences in the definitions and implications of child behavior problems to be largely mediated through caregivers’ influences on the microsystems of child development^23^.

This paper employs the theoretical framework described in Super & Harkness’ developmental niche model. The developmental niche is a model for understanding cultural-ecological influences on the development of emotions and behaviors in child development through examination of cultural regulation of the child’s micro-environment^31^. Their model focuses attention on how physical and social settings, customs of childcare, and caregivers’ ethnotheories interact to create the environment in which children’s development is shaped and culture is passed on. Empirical research based on Super & Harkness’ model has consistently demonstrated that parents’ ethnotheories affect their parenting goals and caregiving strategies to achieve these goals^26,34^. Their work has had a particular focus on socialization of affect and emotion regulation^35^.

We examine gender as a socially constructed framework that—because of its pervasive and largely understudied influence—is important to consider due to its effects on caregivers’ attitudes, beliefs, and behaviors. Gender is viewed as a social construct that is perpetuated through people’s actions and relationships, emerging through social transactions labeled as gendered, rather than an essentialist, static property residing within an individual^36–38^. In their conceptualization of the developmental niche, Super and Harkness identify gender as one of the primary factors that modifies the microsystem of child development. They note that “variations in sex and temperament are among the most evident personal characteristics whose meaning and consequence are organized by features of the developmental niche”^39^. Their observation emphasizes the social construction of gender and suggests the strong influence that meanings of gender have on organizing the settings to which children are exposed, caregiver’s ethnotheories about appropriate behavior and discipline, and the approaches caregivers take to rearing girls versus boys.

## Study Aims

The purpose of this study was to understand how behavioral expectations differed for girls versus boys in the context of culturally-specific gender roles and gender discrimination practices. Our objectives were to identify how gender constructs affect parents’ and teachers’ expectations of children’s behavior in everyday roles, their responses to children’s non-adherence to behavioral expectations (i.e. “behavior problems”), and their understanding of anticipated consequences of behavior problems. We utilized the overarching framework of the developmental niche to guide our analysis, evaluating how gender interacted with settings, caregivers’ ethnotheories, and caregivers’ practices to construct perceived differences in child behavior problems.

## Methods

### Research setting

This study was set in the village of Meghauli in the Chitwan District of the Terai region in south-central Nepal. Meghauli is one of the 40 villages in the Chitwan District. Meghauli has a total population of 14,149. The sex ratio (male:female) in Meghauli is 0.81, largely due to the large number of males living and working elsewhere in Nepal or abroad. The most common language spoken in the region is Nepali, followed by Tharu. The largest caste/ethnic group in the region is Tharu (27% of the population), followed closely by Brahmin (22%). The current literacy rate among women in Meghauli is 68.4% and among men is 82.3%^40^. This village was selected on the basis of its participation in a primary care mental health integrated care delivery project^41,42^. This study was conducted as part of formative research to understand the local context in order to inform interventions.

### Study design

We conducted an exploratory qualitative case study to evaluate how gender affects the definitions and interpretations of and responses to child behavior problems in a small community in rural Nepal^43^. A case study approach was chosen because we were interested in the interrelationships between gender and child behavior problems, but these issues could not be understood outside of the context (i.e. rural community in Nepal)^44^. The purpose of the study was primarily exploratory, as we sought to understand how gender affects local stakeholders’ definitions and interpretations of and responses to behavior problems^43^. The case was bound by a focus on the Meghauli community, specifically children ages 8–15 and behavior problems. The age 8–15 was chosen in order to obtain a larger breadth of understanding regarding the expectations of children and youth in Nepal, and, in order to reflect common ages of school attendance in the local community—where children often do not start school until age seven or eight, and frequently leave school after grade eight^45^. (See Supplementary Table 1 for additional details on study methods using the consolidated criteria for reporting qualitative research (COREQ)^46^).

### Sampling and participants

Participants were purposively sampled on the basis of their familiarity with children and child rearing practices in the community. Local Female Community Health Volunteers (FCHV) helped to identify participants (N = 14) who were selected on the basis of their involvement with children as either teachers/school employees (n = 3), community leaders (n = 5), or parents (n = 6). Stakeholders held roles in varying spaces within the community including government, schools, construction, farm, and the home. Participants came from various ethnicities, educational and socioeconomic backgrounds reflecting the diversity of the Meghauli village (See Table 1). The age of participants was reported in a range to preserve anonymity, and ranged between 25–55 years old, and five out of the 14 participants were women.

**Table 1:** Characteristics of participants in semi-structured interviews.

Characteristic	Number
**Sex**	
Female	5
Male	7
**Age**	
25–29	1
30–39	4
40–49	4
50–60	1
>60	1
N/A	1
**Caste/Ethnicity**	
Brahmin/Cheetri	5
Dalit	1
Janajati	3
Madheshi	1
Tharu	1
N/A	1

Notes: “N/A” = data on age and caste/ethnicity not reported for one participant.

### Ethical considerations

The Johns Hopkins Bloomberg School of Public Health Institutional Review Board and the Nepal Health Research Council reviewed and approved this study before any data collection began. All studies were performed in accordance with the principles outlined in the Declaration of Helsinki. Prior to interviews, all participants provided verbal informed consent. Participants were encouraged to ask questions regarding the study procedures prior to giving consent. Participants were compensated with small household items with an approximate value of US $2–3, as recommended by a Nepalese ethical review board and consultation with local researchers.

### Data collection

Semi-structured interviews were conducted in Nepali using an interview guide (see Supplementary Table 2). Questions focused on: role expectations of children (including a review of children’s daily schedules, from morning until night), definitions of desirable and undesirable behavior, responses to such behavior, situations in which typically undesirable behavior was excused, as well as perceived causes of undesirable behavior. To understand potential mitigating effects of gender and age, participants were asked to describe differences between boys and girls and between younger (eight- to nine-year old) and older (14- to 15-year old) children for each question.

All interviews took place in private convenient locations such as the participant’s home or nearby locations. The interviews were audio-recorded and typically lasted between 30–90 minutes. The interviewer returned to re-interview particularly informative participants multiple times for follow-up questions over the course of 10 months.

### Data preparation

Audio recordings from the interviews were translated and transcribed verbatim and then translated into English. Handwritten notes taken during the interviews (related to nonverbal communication and the physical surroundings) were included in the transcripts in parentheses. Ambiguous key phrases and terms were retained in Nepali in the transcripts and reviewed by an anthropologist and Nepali researcher. The transcripts were spot checked by an anthropologist fluent in both English and Nepali. NVivo software (version 10) was used to store and code the data^46^.

### Data analysis

Interview materials were analyzed by authors J.L. and M.B. using methods drawn from conventional content analysis^47^. We used predetermined and emergent codes to identify expectations, problem behavior, and responses to problem behavior, with each code stratified by gender. These codes were created using open (preliminary) and selective (focused) coding strategies. One author (M.B.) performed initial line-by-line coding of the manuscripts in NVivo^48^. These codes were then reviewed independently by a second author (J.L.), who applied additional specific coding for gender-based differences across the original codes. Disagreements were resolved through discussion between both coding authors. The codes were then arranged into categories of emergent themes through discussion between the two coding authors. A third author (J.R.) who had not been involved in data collection or initial analysis then reviewed the initial thematic assessment; at that time disagreements were again discussed and clarifications were added to the analysis. Finally, all of the data was synthesized into four relevant themes discussed in the following section.

## Results

We identified four major themes related to gender and child behavior in the study interviews: (1) self-reported gender non-bias; (2) differentiated role expectations; (3) gender, “goodness”, and differential thresholds for problem behaviors; (4) boys and girls require different responses for misbehavior. In apparent contradiction, respondents often emphasized similarities by gender earlier in the interview and later discussed day-to-day differences in expectations, responses to, and anticipated consequences from behavior problems by gender.

### Theme 1: Self-reported gender non-bias

Early in the interviews, most (12 out of 14) participants indicated that there was no difference in their expectations for boys and girls or the behaviors they observed between them. The interviewer noted that six stakeholders emphasized that they did not discriminate between girls and boys. One woman, a homemaker, summarized the comments of many of those interviewed, “There is no discrimination between sons and daughters here”.

Participants stated that it was important to them for girls and boys to participate in the same activities and hold similar daily routines. For example, respondents described the expectations of studying and school attendance to be of equal importance for boys and girls. Particularly for younger children, respondents noted similar expectations for boys and girls: to play and to complete their household chores. When asked about younger boys’ daily responsibilities, one participant responded:

*Like the girls after completing 5 years*, *they [boys] wash the dishes after eating and prepare tea when there is guest;* they *clean the house*, *decorate the room*, *wash the clothes; the daughters do this and we expect same from the son*. *-* Female (Homemaker)

To support their views of “no difference” between boys and girls, several respondents highlighted recent governmental policy shifts toward gender equity in land inheritance and school attendance. One male business worker stated, “There is no difference between the son and daughter…. now everyone gets the parental property, as the government has such policy”. Others highlighted the current equality enjoyed by girls by contrasting contemporary expectations with gender differences prevalent during their own childhood. For example:

*There used to be lots of work at home and we did not get time to touch the books*. *When we studied*, *our parents used to scold us saying*, *“Why should a girl study?” But that is not present now*. *We wonder if we* used *to get these facilities at that time*, *then what would our lives be like now?* - Female (Female Community Health Volunteer (FCHV))

### Theme 2: Differentiated role expectations

Following respondents’ more general statements about the similar expectations between boys and girls, they were asked to describe specific roles and expectations of girls and boys. Through this questioning, stakeholders described their daily expectations and how they actually do differ between genders.

Eight participants highlighted differential expectations of boys and girls pertaining to household chores, particularly in food preparation and cleaning. Boys were more often reported to assist with “outside” work and tending to farm animals. A FCHV noted:

*In the home*, *between the son and the daughter*, *the daughter should work in the kitchen*, *as… the daughter should know all the stuffs of the kitchen and … are made to work in the kitchen*. *The son [does]… the outside work like bringing water and feeding the cattle…*- Female (FCHV)

One father commented, while watching his daughter play with a mini kitchen set:

*We consider the girls to take the broom since…birth*. *The girls usually help in cleaning the house*, *other household chores and they study*. *Look at her* (*pointing at his daughter who was playing with a kitchen play set*)*… she is playing with that; if there was a boy*, *he usually plays with a ball*. - Male (Government worker)

Four respondents noted women were responsible for teaching their daughters skills to be a good wife and mother, while men (to a lesser extent) were responsible for teaching their sons to be good husbands. One female FCHV respondent stated, “the girls also learn the work, as they have to go to the other’s [their husband’s] house”. Another male respondent commented on the differing duties between daughters and sons and their relationship to the same-gender parent:

If the son helps in the work of the father, then the daughter should help the mother. If they [daughters] study for one to one and a half hours and then help the mother, then it will be good …and also the burden will be less for the mothers. It will also be practice for the daughters. - Male (Farmer)

Boys were expected to emulate their fathers, but participants expressed more concern with how their daughters fulfilled their daily gender role expectations. A male (health center assistant) noted, “in every condition there is the difference between the activities done by the son and the daughter, there is a big difference”. Participants noted that families’ honor and prestige largely depend on the daughter’s fulfillment of her gender role, and the burden falls to mothers to uphold children’s good behavior. A homemaker remarked “as the mother is in the home so she is the one responsible… because she is home, she should control the children”. A male who was a former school principal remarked, “it is hard to talk about the daughters as the topic is sensitive. For boys it is a minor thing…”

Stakeholders discussed how differing household roles of men and women in the community strongly impacted the expected behaviors of children. Participants expected boys to go to school, to return home, to do school work, and to play. Boys are able to spend time outside running and playing, and fighting with each other. Girls are expected to go to school, come home and work with their mother to run the house. They are given very little ‘play time’ and are expected to practice running a home, often having to fit in their school work between or during household chores.

*In home*, *between the son and the daughter*, *the daughters are made to work in the kitchen*, *as it is thought that the daughter should know all the* stuffs *of the kitchen… the son is asked to do the outside work*, *like bringing the water and feeding the cattle*. - Female (FCHV)

To explain these differences, respondents often referred to traditional roles (including marital roles, as above) and long-held customs. For example, a male (health center assistant) reflected, “Even if there is an elder brother, we ask the daughter to help us, which is the practice from ancient times”.

### Theme 3: Gender, “goodness”, and differential thresholds for problem behaviors

When specifically discussing gender and behavior problems, key themes emerged relating to different underlying “natures,” different thresholds for misbehavior, and different types of behavior considered unacceptable for boys versus girls.

#### “Girls are good:”

Most of the participants (9 out of 14) stated that in their community, girls were inherently “good” (Nepali: *raamro*), that they were helpful contributors to families and communities and did not misbehave, and that they were expected to remain this way. When participants were questioned about misbehavior in girls, a typical response involved describing girls’ generally “helpful,” compliant behavior.

*There is no such problem with the girls; the problem is not at an extreme level in them*. *We have not seen them spoiled*, *destroyed*, *or being with bad company*. *We have seen them helping each* other*…their family*, *parents or the community*. *Like if there is a program and we ask them to do something*, *then they will be prepared and do it and never say no to it…the daughters of the age 14–15 are not that bad*. *They are good*. - Male (Teacher)

Boys were described as having major difficulties conforming to the prosocial behavior expectations of the community. Five of the respondents described boys as being reluctant to perform any of their expected duties. Boys’ reluctance to assist was described by a young female homemaker: “We have to force them [boys] for everything: we have to force them to study, to eat…”

A male construction worker summarized a commonly held sentiment about gender differences in behavior problems: “The problem is in boys. We have not seen such problems in girls”.

#### Different thresholds

Some behaviors were unanimously considered unacceptable by respondents. The respondents identified disruptive behaviors as: disobedience, drug use (especially marijuana) and alcohol use, disregard for or failure to complete academic expectations, involvement with “mischief” (Nepali: *chakchake*), disrespecting authority figures (parents, teachers, elders etc.), and “wandering” through the community. However, five participants indicated that some of these behaviors are typical, expected, and somewhat acceptable in boys, especially when they are able to maintain their academic performance or prepare to earn an income for the future.

While some substance use (especially cigarettes and occasional alcohol use) was seen as “normal” for boys, respondents discussed the expectation that girls not partake in any substance use. One male teacher stated: “[girls] do not drink alcohol in our community, this is not the practice. The daughters are more good than the sons in our community”.

Respondents referred to different types of behaviors as being unacceptable when discussing boys versus girls. When asked what would be considered misbehavior for girls, eight participants pointed out that being “idle” or not fulfilling obligations related to household duties was specifically a problem for girls. A participant noted:

*The daughters of eight to nine years have to do something in the home*, *like they have to wash their clothes… if they do not do this and sit idly and watch television and do not care about their health and cleanliness and instead* would *rather play than concentrate on their cleanliness*, *then they are called bad*. - Male (Teacher)

While this respondent suggested that play kept girls from fulfilling their expectations, other participants suggested that boys had a “need” to play and were encouraged to do so. Overall, participants expected girls to go directly home after school to complete their house and schoolwork.

Parents kept their daughters at home for fear of the community suspecting them to be “wandering” the streets after school hours, which was deemed inappropriate and problematic behavior. Parents reported fears that girls would be perceived as having run away with a boy without her parents’ permission. (“Running away” meant leaving town and living with a boy and often implied sleeping together outside of wedlock.) A male health center assistant stated, “we are worried our daughter will run away with other men… it is not necessary for us to be suspicious after 21–22 years… between 15–21 it is more difficult for daughters and we should care more”. One respondent alluded to the cause of this fear stemming from the impact it could have on the girl’s future marriage potential. A male former school principal stated that “going out (on dates) will directly have an impact on the (perceived) character of the daughter”. Respondents emphasized the importance of reputation for girls, as it can affect her future marriage prospects even in girls as young as eight.

### Theme 4: Boys and girls require different responses for misbehavior

Stakeholders were asked to describe consequences for non-adherence to expectations, or what was defined as misbehaving. Typical responses to misbehavior included scolding children, admonishing them (Nepali: *samjhaune*; literally meaning “reminding”; with regard to children this can mean pointing out what the problem was and describing a more acceptable alternative), and physical punishments (referred to as “beating”). One homemaker, when asked to describe appropriate punishments for children said, “If he does not obey, we should shout at him or beat him, then we call him bad if he does not obey”.

Ten respondents noted that, in recent years, guardians have favored admonishment over beating for boys. There were two primary reasons respondents cited for the declining use of beatings: first, if one simply beats the boys, they will not learn appropriate behavior; and second, respondents believed boys would become accustomed to the pain from beatings and it would no longer serve the desired effect. A female community health volunteer said “The more we beat the children, their body will be more adapted to it and by beating nothing will happen”. Another respondent added that boys were seen as constantly fighting each other so they were assumed to have become desensitized to pain. A participant explained her preference for admonishment:

*Why should we beat them? By beating he does not learn anything*. *Rather than beating him*, *we should admonish him to follow another path*. *He may not do so today*, *[or] tomorrow but he will the next day*. - Female (FCHV)

For girls, punishment had a different reasoning and approach. Participants described girls as being too sensitive to be beaten or even severely scolded. Only five participants included descriptions of punishments for girls, and punishments were described by others as being a very uncommon practice with girls. A female health center assistant and part-time school staff member highlighted the rationale for differential punishment between boys and girls: “Even if we slap boys and shout (at them), they do not obey us, they feel no pain. But, for the girls, even if we talk loud then they start to cry”.

Respondents described girls as not needing punishment because they were inherently “good” and doing bad things was not in their nature. Girls were considered inherently honest, and did not maliciously attempt to break rules or disobey. A Male (health center assistant) said, “We do not beat them [girls] as they are honest”.

Four out of the five participants who discussed punishments for girls reported that girls also have to be scolded and admonished to learn proper behavior and skills. In this case, participants described the rationale for punishment as being important in preparing the girl to leave her natal home and teaching her how to behave appropriately in her marital home. A male former principal suggested that girls have to learn how to follow directions and be obedient; otherwise, their life in the future will be much harder. If they do not learn to take care of their childhood home, they will not be able to take care of their marital home, inferring that this would jeopardize their marriage.

In summary, respondents viewed admonishment as an acceptable and effective tool to teach appropriate behavior for both boys and girls. Beating was described as an acceptable–but not always effective–punishment for boys, whereas many respondents reported that girls did not need to be punished, or that beating or scolding was too harsh of a punishment for the girls.

## Discussion

This qualitative case study in rural Nepal explored how behavioral expectations differed based on gender role, gender inequity, and treatment of children based on their gender identity. More specifically, the study focused on how gender constructs affect parents’ and teachers’ expectations of children’s behavior in everyday roles, their responses to non-adherence to behavioral expectations (i.e. “behavior problems”), and anticipated consequences of behavior problems. We identified four major themes in the interviews: (1) self-reported gender non-bias; (2) differentiated role expectations; (3) gender, “goodness”, and differential thresholds for problem behavior; and (4) boys and girls require different responses for misbehavior. Underlying these findings are widely shared concepts of masculinity and femininity, ethnopsychological concepts of sensitivity to punishment, and an important link between girls’ reputation and her and her family’s future well-being. The findings are reviewed in detail below, along with additional discussion of sociocultural and historical background, study limitations, and implications for practice and future work.

Many of the gender norms applied to girls and women in this study were related to and reinforced by their role as homemaker within a patriarchal society, while boys were given more educational and vocational opportunities by their families and broader social structures^28^. This study finding aligns with a synthesis report of Early Childhood Care and Education initiatives in 11 countries in Asia, including Nepal^49^. On a day-to-day basis from a young age, Nepali girls are expected to do more of the domestic work (5.8 versus 2.8 hours per day in boys), including more strenuous physical labor like collecting firewood, while playing significantly less than boys (30 minutes versus 1.2 hours in boys)^6^. Due to the value placed on their domestic workload, girls have much lower school attendance and completion rates compared with boys^50^. For some girls, in order to escape the intensive workload, they may join the armed forces^19,51^. As a downstream effect of the strained lived experiences of young girls, women are more likely to devote larger portions of their household’s income in the education of their children^52^. Through similar mechanisms, in recent decades, the opportunities afforded to women and girls in Nepal has been rapidly changing^53,54^. These changes have included increasing land ownership among women, decreasing violence against women, providing legal support to girls affected by violence and abuse, and promoting girls’ school attendance and educational outcomes^55–57^. Other patterns including the expected heavy workloads of girls compared with boys, however remain unequal^7,58^. These apparent contradictions occur at the intersection of longstanding culturally-rooted gender norms and globalization with changes in policy and rhetoric. In our study, the tensions between history and recent change were apparent in participants’ oscillating attitudes about equality, on one hand, and ongoing patterns of differential expectations in daily life, on the other.

Another key finding related to how participants discussed, defined, and responded to behavior problems differently in boys compared with girls. Participants in our study openly discussed behavior problems they observed in boys, but only mentioned positive expectations of girls and were reluctant to discuss problematic behavior in girls. They also noted distinct underlying schemas about what kind of responses are desirable and effective for addressing behavior problems in boys compared with girls. Studies in the United States, Taiwan, and South Korea also demonstrated that teachers rate girls higher than boys on behavioral regulation, and given the added pressure on girls to bring honor to their family in Nepal, this study’s results may be seen differently^59^. On the other hand, when girls are able to attend school, and continue to have a similar pressure to perform, they tend to exhibit strengths in advanced social, behavioral, and reading skills^60^. We propose that employing concepts of masculinity and femininity within the Nepali socio-cultural context can help explain the observed differences in expectations and responses of caregivers^45,61^. Thus, below, we examine the influence of broader cultural beliefs on “caregivers’ psychology”–i.e., one of the key components of Super & Harkness’^27^ developmental niche, and a potential mediator transmitting culture into everyday childrearing and child development^27^.

As alluded to above, an organizing theme in many of the interviews related to the anticipated marriage of girls. Participants noted that it is important for girls to fulfill daily role expectations (primarily cooking and cleaning), obey authority, and behave with modesty and temperance. According to respondents, these behaviors of responsibility, obedience, modesty, and temperance, as well as other “feminine” traits—such as sensitivity—were cultivated from a young age. Behavior problems in girls were often defined in contrast to the comportment desirable of an idealized Nepali wife. In other words, to discuss misbehavior in girls would threaten the promise of a successful marriage and thus a family’s future prestige, and financial wellbeing. In contrast, the primary lens for raising boys, was to prepare them for vocational success. For boys, the groundwork for vocational success in adulthood was laid during childhood by promoting educational attainment, exploratory behaviors (e.g. through play), and social connectedness^6,62^. Dominance and aggression were also more desirable among boys, as evidenced by greater tolerance for fighting among boys in contrast to girls. Such behavioral patterns and traits characterized by exploration, achievement, aggression, and dominance have been associated with masculinity both in and outside of Nepal^28,63^. Many prior studies, however, have shown promise in greater opportunities for economic development of nations when metrics of gender equality increase and thus so does the academic and economic performance of girls^60^.

Another key difference was noted in the gendered pattern of rationales for different responses to behavior problems. Girls were seen as highly sensitive, thus harsh verbal or physical punishments was not seen as beneficial or effective. Boys were seen as insensitive to verbal or physical punishments and less affected by fear as a motivator. Some participants noted that boys could become tolerant to beatings, causing physical punishments to lose their effectiveness. These differences in sensitivity to punishment appear to be rooted in the Nepali ethnopsychological concept of the “heart-mind” (Nepali: *man*), with girls having a “soft/sensitive heart-mind” (Nepali: *kamalo man*), as previously elaborated by Kohrt and Maharjan^45^.

While beatings were still reported to be used for boys, admonishment (Nepali: *samjhaune*) was seen as an effective option for addressing behavior problems in both boys and girls. Admonishment was described as a discussion between an elder and a child, usually focusing on evaluating possible negative future outcomes of continuing an undesirable behavior (or “path”)^62,63^. Respondents admonished girls, for example, by leveraging the fear of harming future marriage prospects or their family’s reputation, which motivated girls to fulfill daily tasks and avoid behaviors identified as problematic. In girls, admonishment was advocated as the softer alternative to punishment required by girls’ high sensitivity; in boys, admonishment was used due to their low sensitivity to physical punishment. Again, relating to previously described patterns of masculinity and femininity in which boys and men are assumed to be rational, assertive, and strong, and girls and women are assumed to be emotional, passive, and weak^64–67^.

As an urban, educated, high-caste woman, the interviewer’s identity may have introduced social desirability bias in participants’ responses. Desirability bias and reflexivity could help explain the initial denial of gender biases, especially in the context of changing national rhetoric surrounding gender equality. Alternatively, the types of questions asked may have elicited different response patterns through the course of the interviews. For example, direct questions about gender differences elicited more proclamations of gender non-bias compared to shared anecdotes about general daily activities during later parts of the interview. And lastly, participants may have been hesitant to share openly about girls due to the impact that negative reputations of girls and women have on the honor or prestige of a village. An additional limitation of this study was the small sample size that originated from a single community. Therefore, our findings are unlikely to be generalizable to the perspectives of people in other regions of Nepal. This study also employed the theoretical binary construct of gender, leaving little room for alternative gender systems to be adequately explored in this Nepali context.

The stakeholder perspectives identified in this study demonstrated a gender-informed discrepancy that aligns with previous epidemiologic studies finding higher rates of disruptive behavior in boys compared with girls^1,2,68,69^. However, our study provides additional cultural context for differential rates of behavior problems and suggests mechanisms through which these differences emerge. Our findings suggest that cultural norms do not allow girls to be disruptive, whereas boys have the opportunity, cultural space, and expectation to conduct themselves in a disruptive manner^70–72^. Previous studies of gender differences in behavior problems have primarily been quantitative epidemiologic studies reporting differences in rates of specific behaviors and behavior problem-related disorders^1,2^. Our study furthers previous epidemiologic studies by drawing upon a constructivist epistemology and social interactionism theoretical framework to investigate the social construction of gendered differences in behavioral expectations and responses to problematized behaviors. Specifically, we investigated three under-studied components in gender differences in behavior problems: (1) gender differences in expectations of child behavior, (2) the differential significance of specific behavior problems depending on the child’s gender, and (3) differential responses (i.e. consequences) for specific behavioral expressions depending on the child’s gender. The combination of these differences in expectations, meaning, and consequences is likely to have implications for interpreting findings from the aforementioned epidemiologic differences (i.e. different rates of behavior problems by gender) rooted in objectivist epistemologies.

Our findings can be used to inform the adaptation and implementation of interventions targeting problematic behaviors and mental health and wellness in Nepalese children. Greater understanding of the differential expectations and responses to disruptive behaviors by gender is necessary to understand, identify and later address behavior problems or other mental health difficulties in this context^18^. Our findings can be introduced into discussions about gender equity in childhood as an important determinant of present and future physical and mental health^7,73,74^. Our study provides a call to better empower caregivers to reflect on gender norms and how they influence the behaviors and thus overall developmental trajectory of children^75^. Equity is especially important to consider in strategies to prevent high rates of internalizing disorders among women globally, and high rates of suicide among women in South Asia^76–79^. Initiatives in Nepal promoting gender equity must move beyond rights attainment for girls and women and begin to address interpersonal relationships and the social environments in which gender norms take shape and are perpetuated^12,49^.

## Supplementary information

Supplementary Tables

## References

[1] Merikangas KR (2010). Lifetime Prevalence of Mental Disorders in USAdolescents: Results from the National Comorbidity Survey Replication–Adolescent Supplement (NCS-A). J Am Acad Child Adolesc Psychiatry..

[2] Moffitt TE (2002). Sex differences in antisocial behavior: Conduct disorder, delinquency and violence in the Dunedin Longitudinal Study. Journal of Child Psychology and Psychiatry.

[3] Gaines AD (1992). From DSM-I to III-R; voices of self, mastery and the other: A cultural constructivist reading of U.S. psychiatric classification. Social Science & Medicine.

[4] Diagnostic and Statistical Manual of Mental Disorders, 5th Edition, 10.1176/appi.books.9780890425596.893619 (2013).

[5] Kohrt, B. A. Child Maltreatment and Global Health: Biocultural Perspectives. *Handbook of Child Maltreatment Child Maltreatment* 553–577, 10.1007/978-94-007-7208-3_30 (2013).

[6] Yamanaka M, Ashworth A (2002). Differential workloads of boys and girls in rural Nepal and their association with growth. American Journal of Human Biology.

[7] Bennett, L. Gender, Caste, and Ethnic Exclusion in Nepal: Following the Policy Process and Analysis to Action. *Paper presented at World Bank Conference on New Frontiers of Social Policy: Development in a Globalized World*, Arusha (2005).

[8] Heise, L. L. & Kotsadam, A. Cross-national and multilevel correlates of partner violence: an analysis of data from population-based surveys. *The Lancet Global Health* **3** (2015). 10.1016/S2214-109X(15)00013-3 26001577

[9] Peacock D, Barker G (2014). Working with Men and Boys to Prevent Gender-based Violence. Men and Masculinities.

[10] Eagly AH, Makhijani MG, Klonsky BG (1992). Gender and the evaluation of leaders: A meta-analysis. Psychological Bulletin.

[11] Rudman LA, Glick P (1999). Feminized management and backlash toward agentic women: The hidden costs to women of a kinder, gentler image of middle managers. Journal of Personality and Social Psychology.

[12] Blum, R. W., Mmari, K. & Moreau, C. It Begins at 10: How Gender Expectations Shape Early Adolescence Around the World. *Journal of Adolescent Health* **61** (2017). 10.1016/j.jadohealth.2017.07.009 PMC5612023 28915989

[13] Ollendick TH, Yang B, Dong Q, Xia Y, Lin L (1995). Perceptions of fear in other Children and adolescents: The role of gender and friendship status. Journal of Abnormal Child Psychology.

[14] Trillo VM, Rendo LM (2013). The role of gender identity in adolescents’ antisocial behavior. Psicothema.

[15] Vogel DL, Heimerdinger-Edwards SR, Hammer JH, Hubbard A (2011). “Boys dont cry”: Examination of the links between endorsement of masculine norms, self-stigma, and help-seeking attitudes for men from diverse backgrounds. Journal of Counseling Psychology.

[16] Bell (2014). Adolescent and Young Adult Male Health: A Review. Pediatrics. 2013; 132(3):535–546. Pediatrics.

[17] New Delhi: World Health Organization, Regional Office for South-East Asia. Adolescent Sexual and Reproductive Health Programme to Address Equity, Social Determinants, Gender and Human Rights in Nepal, Report of the Pilot Project (2017).

[18] Rai, S., Adhikari, S. B., Acharya, N. R., Kaiser, B. N. & Kohrt, B. A. Elucidating adolescent aspirational models for the design of public mental health interventions: a mixed-method study in rural Nepal. *Child and Adolescent Psychiatry and Mental Health* **11** (2017). 10.1186/s13034-017-0198-8 PMC5740935 29299056

[19] Kohrt BA (2016). Recruitment of child soldiers in Nepal: Mental health status and risk factors for voluntary participation of youth in armed groups. Peace and Conflict: Journal of Peace Psychology.

[20] Ministry of Health, Nepal. Full Report of Nepal Demographic and Health Survey (NDHS) 2016 (2017).

[21] Ministry of Education, Nepal. Education for all: National Plan of Action 2001–2015 (2006).

[22] Kohrt BA, Jordans MJ, Koirala S, Worthman CM (2014). Designing mental health interventions informed by child development and human biology theory: A social ecology intervention for child soldiers in Nepal. American Journal of Human Biology.

[23] Worthman CM (2010). The Ecology of Human Development: Evolving Models for Cultural Psychology. Journal of Cross-Cultural Psychology.

[24] Kohrt BA, Bourey C (2016). Culture and Comorbidity: Intimate Partner Violence as a Common Risk Factor for Maternal Mental Illness and Reproductive Health Problems among Former Child Soldiers in Nepal. Medical Anthropology Quarterly.

[25] Kleinman AM (1977). Depression, somatization and the “new cross-cultural psychiatry”. Social Science & Medicine (1967).

[26] Harkness, S., & Super, C. M. Parents cultural belief systems: Their origins, expressions, and consequences. New York: Guilford Press (1996).

[27] Super CM, Harkness S (1986). The Developmental Niche: A Conceptualization at the Interface of Child and Culture. International Journal of Behavioral Development.

[28] United Nations Development Programme. Nepali Masculinities and Gender-Based Violence. United Nations Development Programme, New York, NY (2014).

[29] Cameron MM (2009). Gender, Science, and Indigenous Medicine: Planning Research on Asian Women Professional Providers. Health Care for Women International.

[30] Kohrt BA (2010). Social Ecology of Child Soldiers: Child, Family, and Community Determinants of Mental Health, Psychosocial Well-being, and Reintegration in Nepal. *Transcultural*. Psychiatry.

[31] Rani M, Bonu S (2008). Attitudes Toward Wife Beating. Journal of Interpersonal Violence.

[32] Crotty, M. *The foundations of social research meaning and perspective in the research process*. (SAGE, 2015).

[33] Kanter RM, Blumer H (1971). Symbolic Interactionism: Perspective and Method. American Sociological Review.

[34] Harkness S (2007). Cultural models and developmental agendas: Implications for arousal and self-regulation in early infancy. Journal of Developmental Processes..

[35] Harkness S, Super CM (1983). The cultural construction of child development: A framework for the socialization of affect. Ethos..

[36] Gerson JM, Peiss K (1985). Boundaries, Negotiation, Consciousness: Reconceptualizing Gender Relations. Social Problems..

[37] West C, Zimmerman DH (1987). Doing gender. Gender & society..

[38] Bohan JS (1993). Regarding gender: essentialism, constructionism and feminist psychology. Psychology of Women Quarterly..

[39] Super CM, Harkness S (2002). Culture Structures the Environment for Development. Human Development..

[40] Central Bureau of Statistics. National population and Housing Census 2011 (Village Development Committee/ Municipality) (2012).

[41] Jordans, M. J. D., Luitel, N. P., Pokhrel, P. & Patel, V. Development and pilot testing of a mental healthcare plan in Nepal. *British Journal of Psychiatry*. **208** (2016). 10.1192/bjp.bp.114.153718 PMC4698553 26447173

[42] Lund, C. *et al*. PRIME: A Programme to Reduce the Treatment Gap for Mental Disorders in Five Low- and Middle-Income Countries. *PLoS Medicine*. **9** (2012). 10.1371/journal.pmed.1001359 PMC3531506 23300387

[43] So S (2011). Case Study Research: Design and Methods by YIN, ROBERT K. The Modern Language Journal..

[44] Baxter P, Jack S (2008). Qualitative Case Study Methodology: Study Design and Implementation for Novice Researchers. The Qualitative Report..

[45] Kohrt BA, Maharjan SM (2009). When a child is no longer a child: Nepali ethnopsychology of child development and violence. Studies in Nepali History and Society..

[46] Tong A, Sainsbury P, Craig J (2007). Consolidated Criteria for Reporting Qualitative Research (COREQ): a 32-Item Checklist for Interviews and Focus Groups. International Journal for Quality in Health Care..

[47] Hsieh HF, Shannon SE (2005). Three approaches to qualitative content analysis. Qualitative Health Research..

[48] NVivo Qualitative Data Analysis Software. QSR International Pty Ltd. Version 10 (2014).

[49] Plan International. Synthesis Report: Research into gender equality and early childhood development in eleven countries in Asia (2017).

[50] Fixing the Broken Promise of Education for All: Findings from the Global Initiative on Out-of-School Children (2015).

[51] Stevens AJ (2014). The invisible soldiers: understanding how the life experiences of girl child soldiers impacts upon their health and rehabilitation needs. Archives of Disease in Childhood..

[52] United Nations, Economic and Social Commission for Asia and the Pacific. Gender, the Environment and Sustainable Development in Asia and the Pacific (2017)

[53] Kondos V (1991). Subjection and the Ethics of Anguish: The Nepalese Parbatya Parent-Daughter Relationship. Contributions to Indian Sociology.

[54] Levine NE (1982). Social Structure, Fertiity and the Value of Children in Northwestern Nepal. Contributions to Nepalese Studies..

[55] Blumer, H. Symbolic interactionism: Perspective and method. *University of California Press* (1986)

[56] Annan, K. Review of the implementation of the Beijing Platform for Action and Women 2000: Gender, Equality. Development and Peace for the 21st Century, E/CN. 6/2004 (2005).

[57] Pennells, L. Case Study Nepal. Girls’ and Women’s education systems: policy and implementation. *UNESCO* (1998).

[58] Balakrishnan, R., & Fairbairn-Dunlop, P. Rural women and food security in Asia and the Pacific: Prospects and paradoxes. *UN-FAO*. RAP (2005).

[59] Wanless SB (2013). Gender differences in behavioral regulation in four societies: the United States, Taiwan, South Korea, and China. Early Childhood Research Quarterly.

[60] Weber, A., Darmstadt, G. & Rao, N. Gender Disparities in Child Development in the East Asia-Pacific Region: a Cross-Sectional, Population-based, Multicountry Observational Study. *Lancet Child Adolesc Health* (2017). 10.1016/S2352-4642(17)30073-1 30169170

[61] Chaplin TM, Cole PM, Zahn-Waxler C (2005). Parental socialization of emotion expression: gender differences and relations to child adjustment. Emotion..

[62] Burkey MD (2016). The ecocultural context and child behavior problems: A qualitative analysis in rural Nepal. Social Science & Medicine..

[63] Burkey, M. D., *et al*. Roles of Cultural Context in Definitions of and Responses to Conduct Problems in Children: A Case Study from Nepal. *Society for the Study of Psychiatry and Culture* (2015).

[64] Best, S., & Kellner, D. Postmodern theory: Critical interrogations. *Guilford Press* (1991).

[65] Archer J (2004). Sex differences in Aggression in Real-World Settings: a Meta-Analytic Review. Review of General Psychology..

[66] Eagly, A. H., Wood, W., & Diekman, A. B. Social Role Theory of Sex Differences and Similarities: A current appraisal. The developmental social psychology of gender 123–174 (2000)

[67] McCarthy PL (2000). Race/ethnicity, Gender, Socioeconomic Status Research Exploring Their Effects on Child Health: A Subject Review. Pediatrics..

[68] Adhikari RP (2015). Perceived Behavioral Problems of School aged Children in Rural Nepal: a Qualitative Study. Child and Adolescent Psychiatry and Mental Health..

[69] Zeman J, Shipman K, Suveg C (2002). Anger and sadness regulation: Predictions to Internalizing and Externalizing Symptoms in Children. Journal of Clinical Child and Adolescent Psychology..

[70] Keenan K, Loeber R, Green S (1999). Conduct disorder in girls: A review of the literature. Clinical Child and Family Psychology Review..

[71] Eisenberg N (2001). The relations of regulation and emotionality to children’s externalizing and internalizing problem behavior. Child Development..

[72] Ojha SP, Jasmin M, Chapagain M, Tulachan P (2013). Educational and Behavioural Problems among Sheltered Homeless. Children. J Nepal Med Assoc..

[73] Rahman MS, Nahar L (2013). Aggression in boys and girls as related to their academic achievement and residential background. Psychology..

[74] Carter R, Silverman WK, Jaccard J (2011). Sex Variations in Youth Anxiety Symptoms: Effects of Pubertal Development and Gender Role Orientation. Journal of Clinical Child & Adolescent Psychology..

[75] Basu S. *et al*. Learning to be Gendered: Gender Socialization in Early Adolescence Among Urban Poor in Delhi, India and Shanghai, China. *J Adolesc Health*. **61** (2017). 10.1016/j.jadohealth.2017.03.012 28915988

[76] Mahalingam R, Haritatos J, Jackson B (2007). Essentialism and the Cultural Psychology of Gender in Extreme Son Preference Communities in India. Am J Orthopsychiatry..

[77] Mahalingam R, Jackson B (2007). Idealized Cultural Beliefs About Gender: Implications for Mental Health. Soc Psychiatry Psychiatr Epidemiol..

[78] Kohrt BA, Worthman CM (2009). Gender and anxiety in Nepal: The Role of Social Support, Stressful Life Events, and Structural Violence. CNS Neuroscience & Therapeutics..

[79] Hagaman AK, Khadka S, Lohani S, Kohrt B (2017). Suicide in Nepal: a Modified Psychological Autopsy Investigation from Randomly Selected Police Cases Between 2013 and 2015. Soc Psychiatry Psychiatr Epidemiol..

