# Supplementary material for: Gender and Child Behavior Problems in Rural Nepal: Differential Expectations and Responses
Source: Sci Rep. 2019 May 21;9:7662. doi: 10.1038/s41598-019-43972-3 (PMC6529428; doi:10.1038/s41598-019-43972-3)
Supplement: Supplementary file 1 — Supplementary Tables [file 41598_2019_43972_MOESM1_ESM.pdf]

Title: Gender and Child Behavior Problems in Rural Nepal: Differential Expectations and Responses

Authors: Julia A. Langer*[1], Julia V. Ramos*[2], Lajina Ghimire[3], Sauharda Rai[3,4], Brandon A. Kohrt[5], Matthew D. Burkey[1,6]

Author Affiliations

1. Johns Hopkins Bloomberg School of Public Health
2. Johns Hopkins University School of Medicine
3. Transcultural Psychosocial Organization—Nepal
4. University of Washington, Jackson School of International Studies
5. Department of Psychiatry and Behavioral Sciences, The George Washington University
6. Department of Psychiatry, University of British Columbia

Supplemental Table 1. Consolidated criteria for reporting qualitative research (COREQ)[1]

Item	Description
**Domain 1: Research team and reflexivity**	
Interviewer: which author(s) conducted interviews	LG and MB
Credentials: researcher's credentials	JL, MHS; JR, BS; LG, BScN, MA; SR, MA; BAK, MD, PhD; MB, MD, PhD, MPH
Author occupation at the time of study	JL, masters student researcher; JR, medical student; LG, research assistant; SR, research associate; BAK, assistant professor of ; MB, assistant (full-time medical school research faculty), PhD candidate;
Gender of interviewer(s)	Male and Female
Experience and training of authors	JL, MHS in mental health; JR, undergraduate degree in women's studies, medical student; LG, four years' experience in community-based research and health programming; SR, five years of experience with qualitative research; BAK, PhD in anthropology with training in qualitative methods, ethnography, and structured elicitation tasks, and 20 years of experience in qualitative and mixed-methods research; MB, PhD courses in qualitative research, 8 years' experience in clinical and qualitative research
Was a relationship with participants established prior to study commencement	Participants were introduced to the research team through community liaisons (female community health volunteers) who are health workers and known to the study team
What did the participants know about the researchers?	Participants were aware that the research team had conducted prior mental health programs in the community and were researchers based in Kathmandu and the United States
What characteristics were reported about the interviewer(s)?	Organizational affiliation
**Domain 2: Study Design**	
Methodological orientation underpinning the study?	Qualitative case study framework with anthropological structured elicitation tasks (daily schedule). Qualitative analysis was guided by methods from conventional content analysis.
How were participants selected?	Nominated by local community liaison (female community health volunteer) on the basis of their familiarity with children and child-rearing practices.
How were the participants approached?	Approached by local community liaison and study interviewer in their homes and told about the study. Description of the study, study procedures, and consent materials were used as per the protocol approved by the Institutional Review Board.
What was the sample size?	14
How many people refused participation?	0
Where was the data collected	Meghauli, Chitwan District, Nepal
Was anyone else present besides the participants and researchers?	A private location was sought for the interviews. However, for a small number of interviews another family member was present.
What are the important characteristics of the sample?	Participants were recruited based on the basis of their familiarity with children and child-rearing practices in the local context. We particularly sought out participants who were parents, school staff (teachers, principals, and other staff), health workers (especially female community health volunteers--a government-organized role in communities throughout Nepal), and other community leaders

	nominated by the community liaisons. We specifically sought to include representatives from both genders, a variety of castes, and various socioeconomic positions. The age of participants ranged between 25-55 years old, and five out of the 14 participants were women. Participants reported their caste as: Brahmin/Cheetri (5), Dalit (1), Janajati (3), Madheshi (1), Tharu (1), and one was not reported.
Were questions, prompts, guides provided by the authors? Was it piloted?	Semi-structured interview guides (including questions and prompts) were used; piloting was conducted
Were repeat interviews carried out?	No
Did the research use audio or visual recording	Yes, audio recordings were used
Were field notes made during the interview?	Yes, interviewers took field notes after the interview to record non-verbal expressions and any interruptions during the interview
What was the duration of the interview?	Interviews ranged from 30 minutes to 2 hours
How was data saturation ensured?	Review of field notes by research assistants and study supervisors
Were transcripts returned to participants for comment and/or correction	No
**Domain 3: Analysis and Findings**	
How many data coders coded the data?	Two coders were assigned to code the data
How was coding applied?	Coding was done in NVivo 12. Codes were applied via line-by-line reviewing by one author (MB) and was reviewed by a second author (JL).
Were themes identified in advance or derived from the data?	Basic codes were identified in advance according to the primary research questions. Additional emergent codes were derived from data.
What software was used to manage and analyze data?	NVivo 12
Did participants provide feedback on the findings?	No
Were quotations presented to illustrate findings? Was each quotation identified?	Quotations are presented to illustrate findings. Quotes are not identified by name but are identified by the speaker's gender and key role characteristics.
Was there consistency between the data presented and the findings?	Quotations and data presented were compared with reported findings by authorship team
Were major themes clearly presented in the findings?	Four major themes are presented at the beginning of the Results section and as sub-headings in the paper. Each theme is linked to original quotations.
Is there a description of diverse cases or discussion of minor themes?	Minor themes are presented descriptively within narrative descriptions. Where applicable, we also included diverse or exceptional cases.

1. From the COREQ guidelines: Tong A, Sainsbury P, Craig J. Consolidated criteria for reporting qualitative research (COREQ): a 32-item checklist for interviews and focus groups. International journal for quality in health care. 2007 Dec 1;19(6):349-57.

Supplementary Table 2: Key Informant Interview Guide

Stem Question	Probing Questions	Goal
1. What are children between the ages of 8-15 expected to do?	1. How are these children expected to contribute to the family?<br>2. What are they expected to do in school?<br>3. How does this differ for boys vs. girls?<br>4. What is expected of children from high castes? Low castes?	To identify general role expectations for children in the study age range (and variations dependent upon gender, caste, and other key dimensions of variability).
2. How are children ages 8-15 expected to act as they go through the day?	1. What behavior is expected from children age 8-15 compared with younger children? Compared with older children?<br>2. How are children expected to act at school? At home? In the community?	To identify expectations for behavior during normal development in children of the study age.
3. Are there times it is less of a problem for children to behave badly or break the rules (above)?	1. What makes it okay to do (fill in) sometimes and not others?	To identify norms about expected/acceptable types of non-pathological behavior problems anticipated during the study age.
